# Supplementary material for: A comprehensive analysis of the oncogenic and prognostic role of TBC1Ds in human hepatocellular carcinoma
Source: PeerJ. 2024 May 14;12:e17362. doi: 10.7717/peerj.17362 (PMC11100476; doi:10.7717/peerj.17362)
Supplement: Supplemental Information 1 [file peerj-12-17362-s001.zip › Blot/peerj-93217-Raw_data.pptx]

## Slide 1
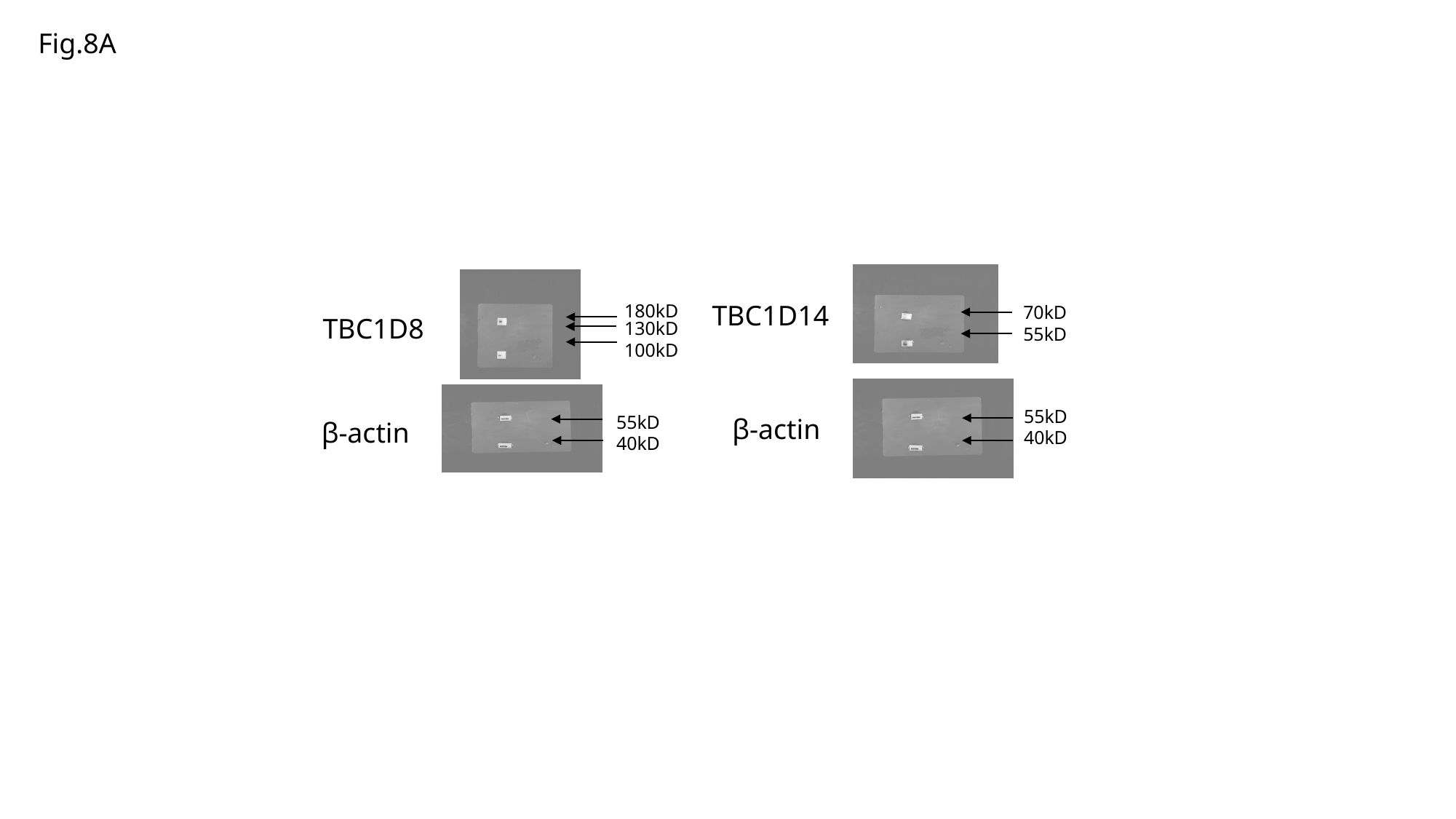

Fig.8A
TBC1D14
180kD
70kD
TBC1D8
130kD
55kD
100kD
55kD
55kD
β-actin
β-actin
40kD
40kD

## Slide 2
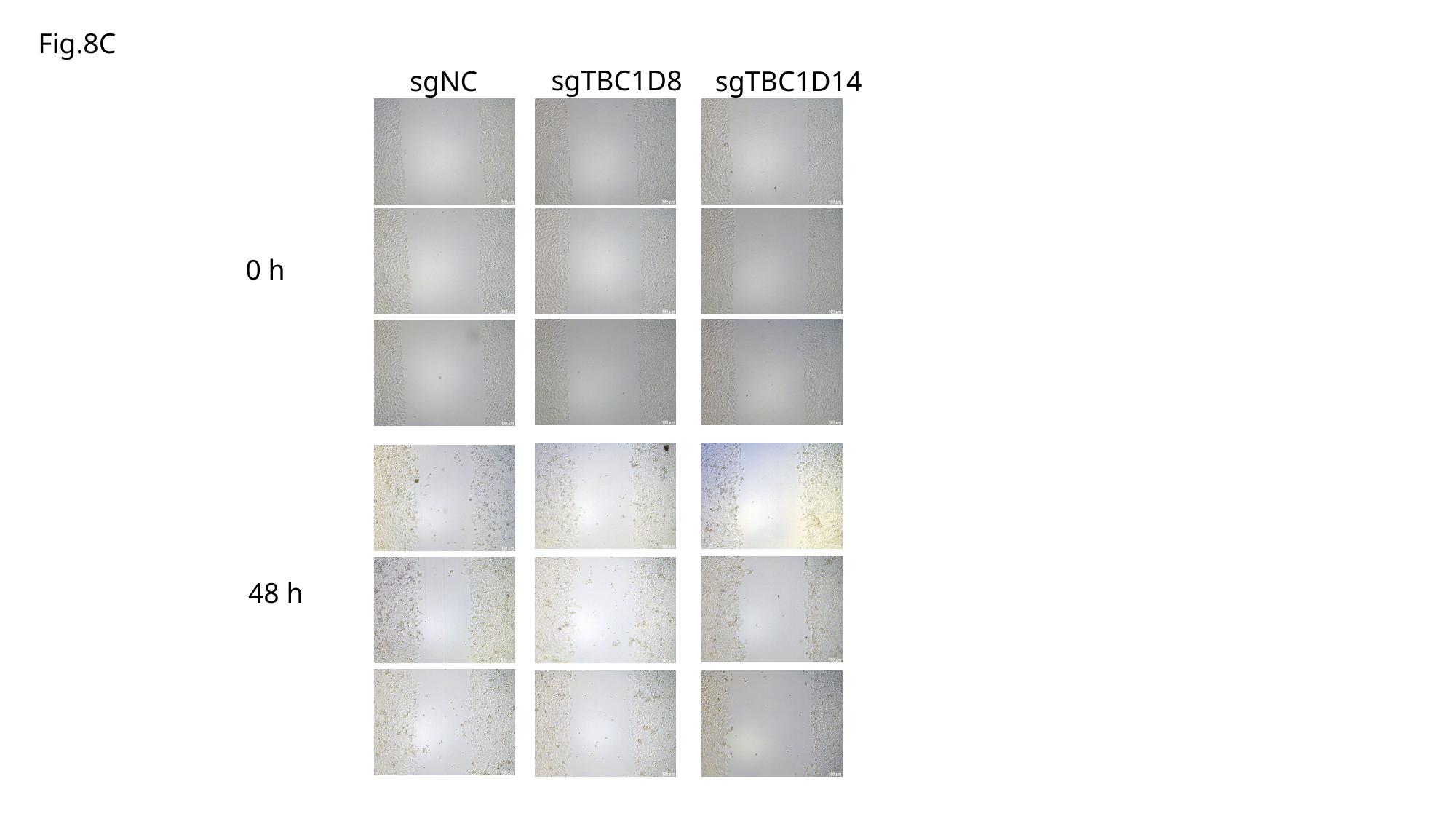

Fig.8C
sgTBC1D8
sgTBC1D14
sgNC
0 h
48 h

## Slide 3
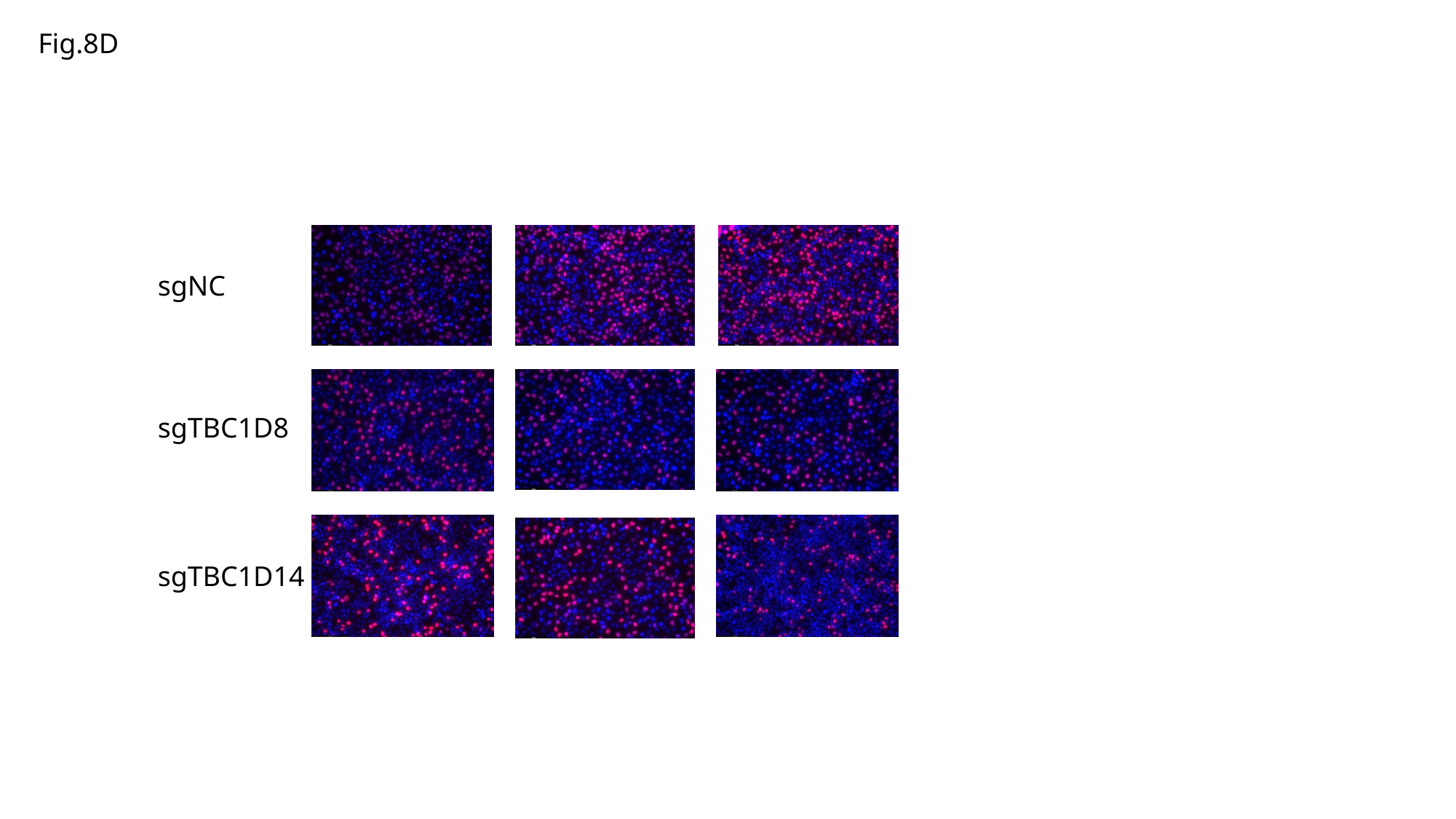

Fig.8D
sgNC
sgTBC1D8
sgTBC1D14
