# Supplementary figures and images for: A comprehensive analysis of the oncogenic and prognostic role of TBC1Ds in human hepatocellular carcinoma
Source: PeerJ. 2024 May 14;12:e17362. doi: 10.7717/peerj.17362 (PMC11100476; doi:10.7717/peerj.17362)

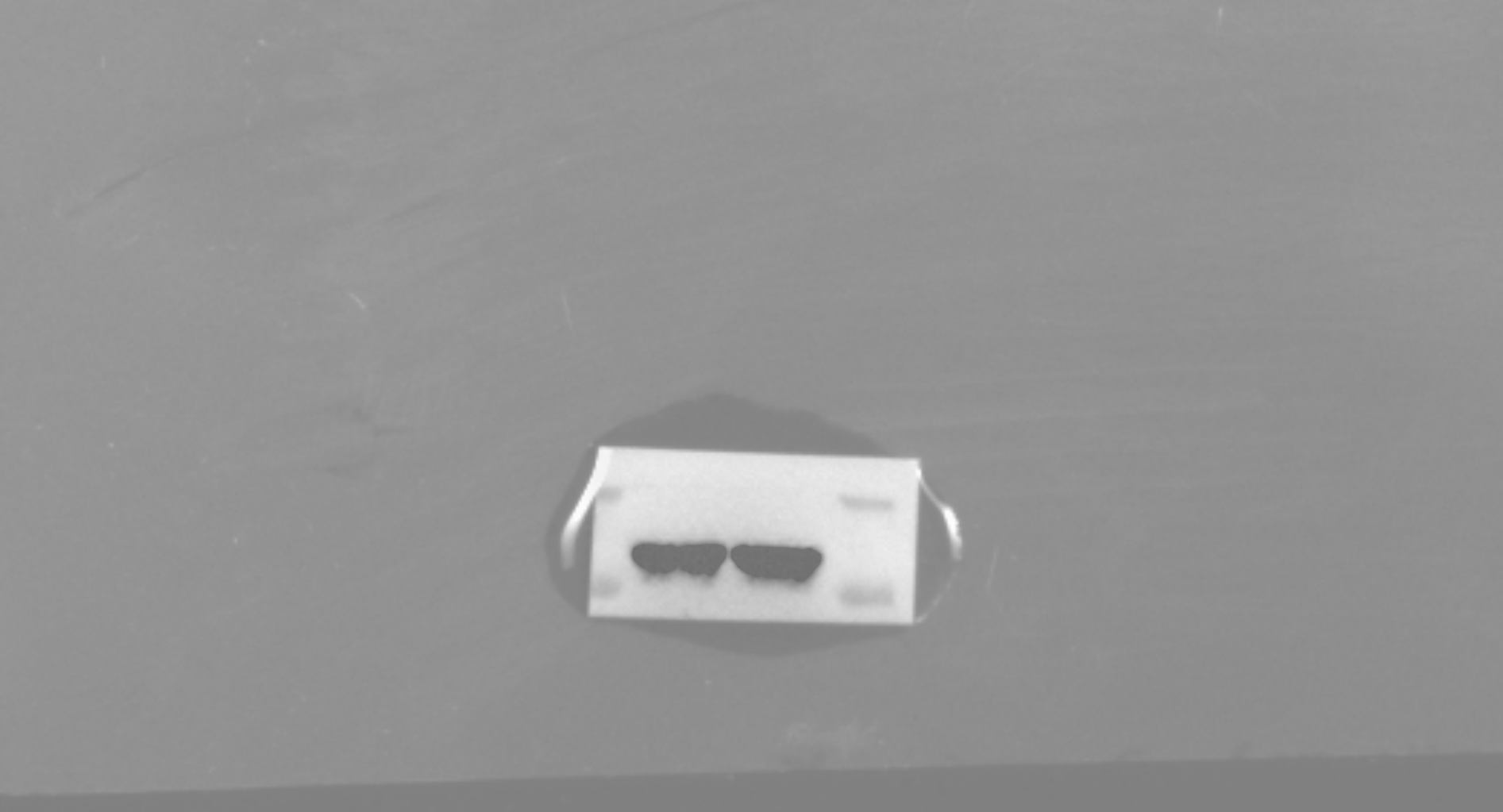

Supplement: Supplemental Information 1 [file peerj-12-17362-s001.zip › Blot/ACTIN-1.tif]

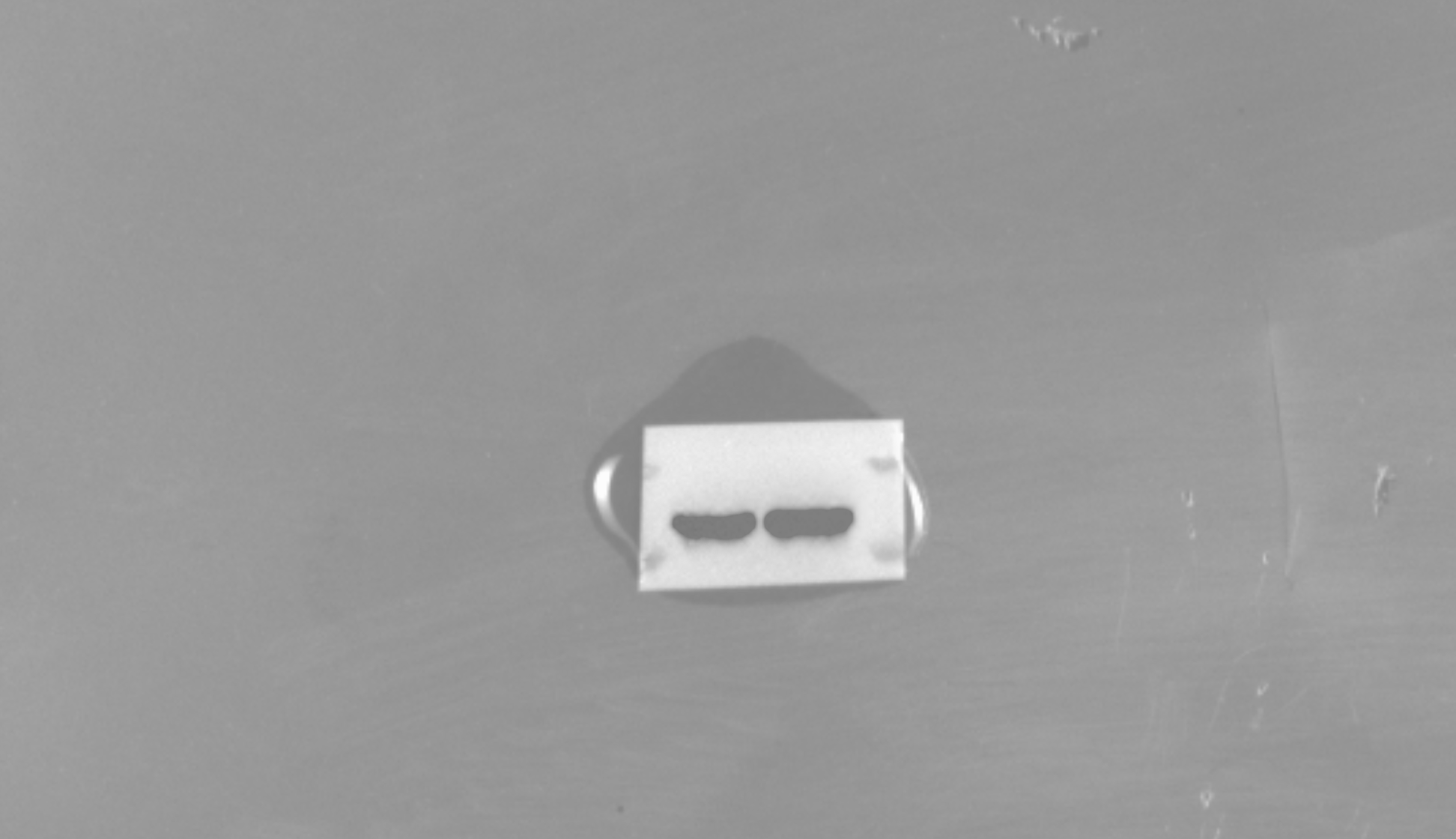

Supplement: Supplemental Information 1 [file peerj-12-17362-s001.zip › Blot/ACTIN-2.tif]

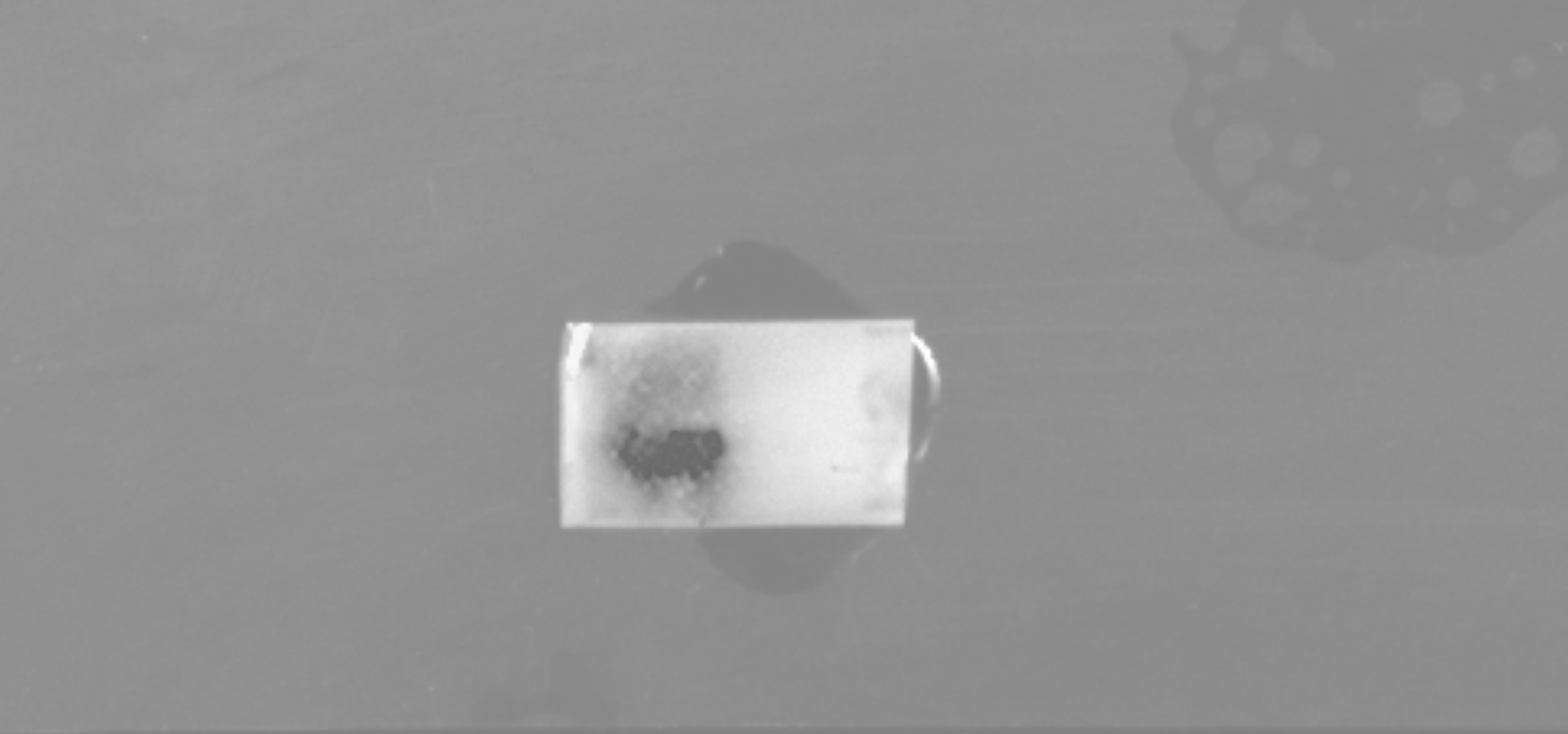

Supplement: Supplemental Information 1 [file peerj-12-17362-s001.zip › Blot/TBC1D14.tif]

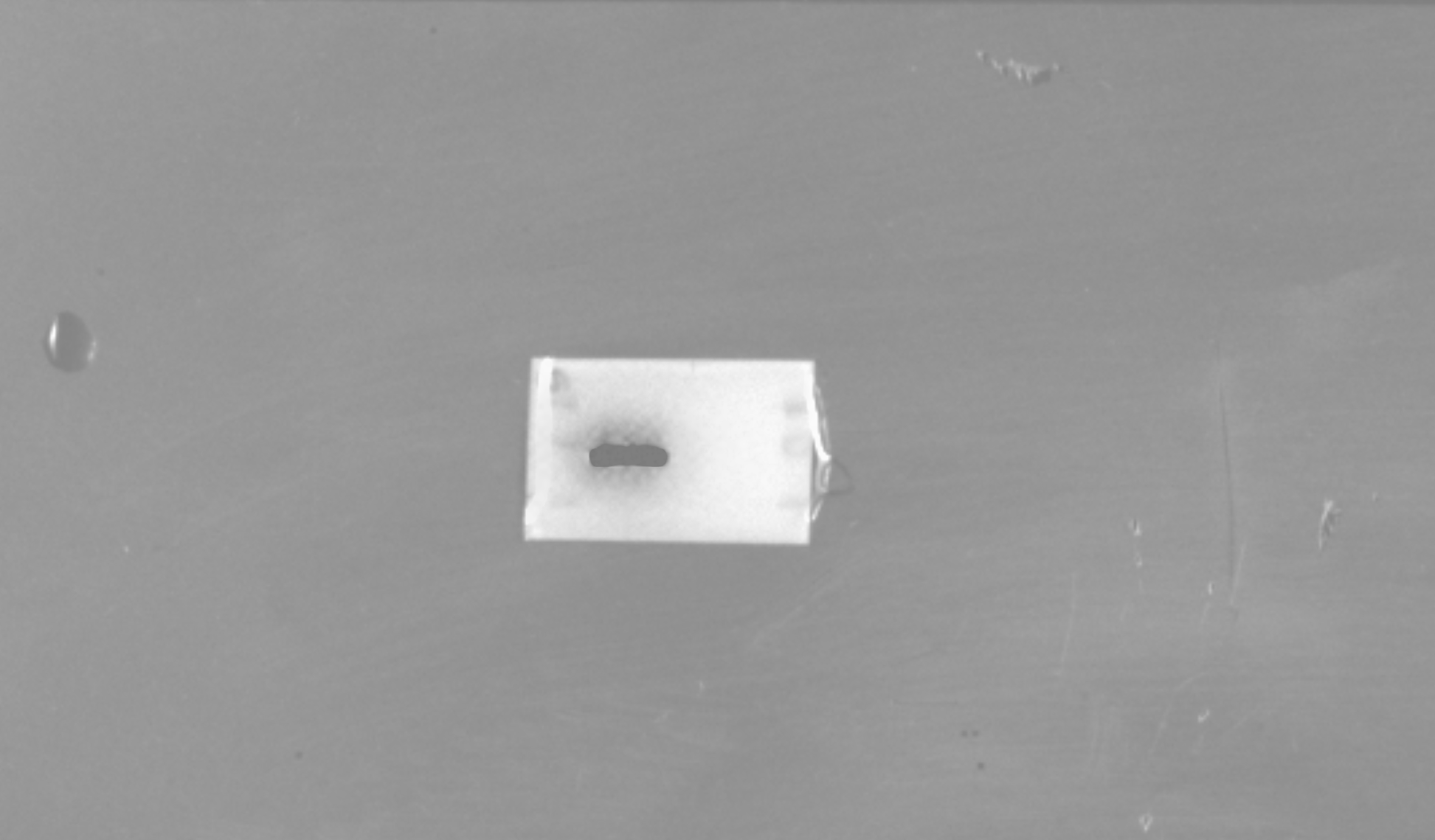

Supplement: Supplemental Information 1 [file peerj-12-17362-s001.zip › Blot/TBC1D8.tif]
