# Supplementary material for: A comprehensive analysis of the oncogenic and prognostic role of TBC1Ds in human hepatocellular carcinoma
Source: PeerJ. 2024 May 14;12:e17362. doi: 10.7717/peerj.17362 (PMC11100476; doi:10.7717/peerj.17362)
Supplement: Supplemental Information 3 [file peerj-12-17362-s003.pdf]

Supporting Figures:

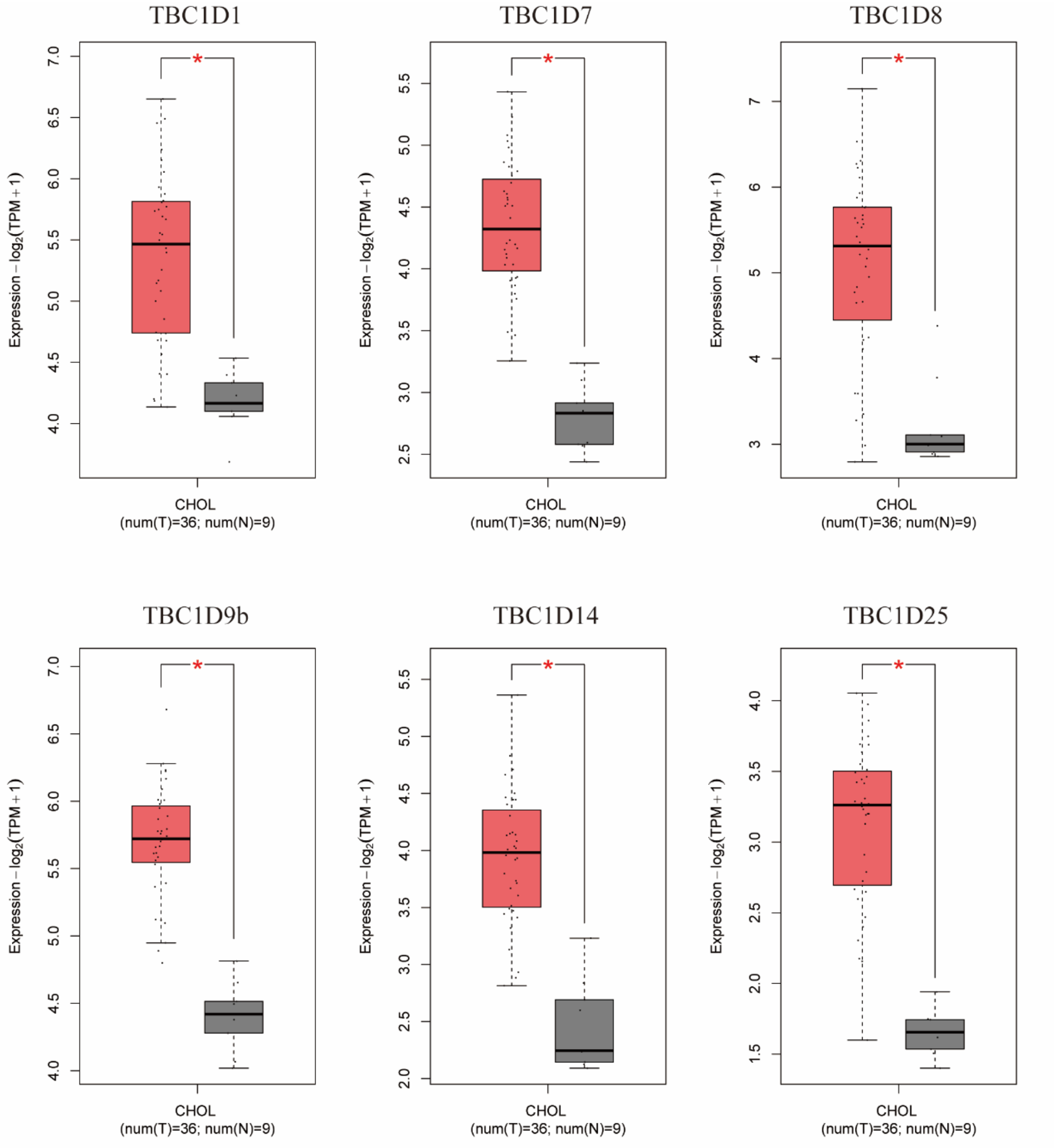

Supporting Figure 1. Transcript level of TBC1Ds in cholangiocarcinoma and paratumor tissues according to the t-test in GEPIA database.

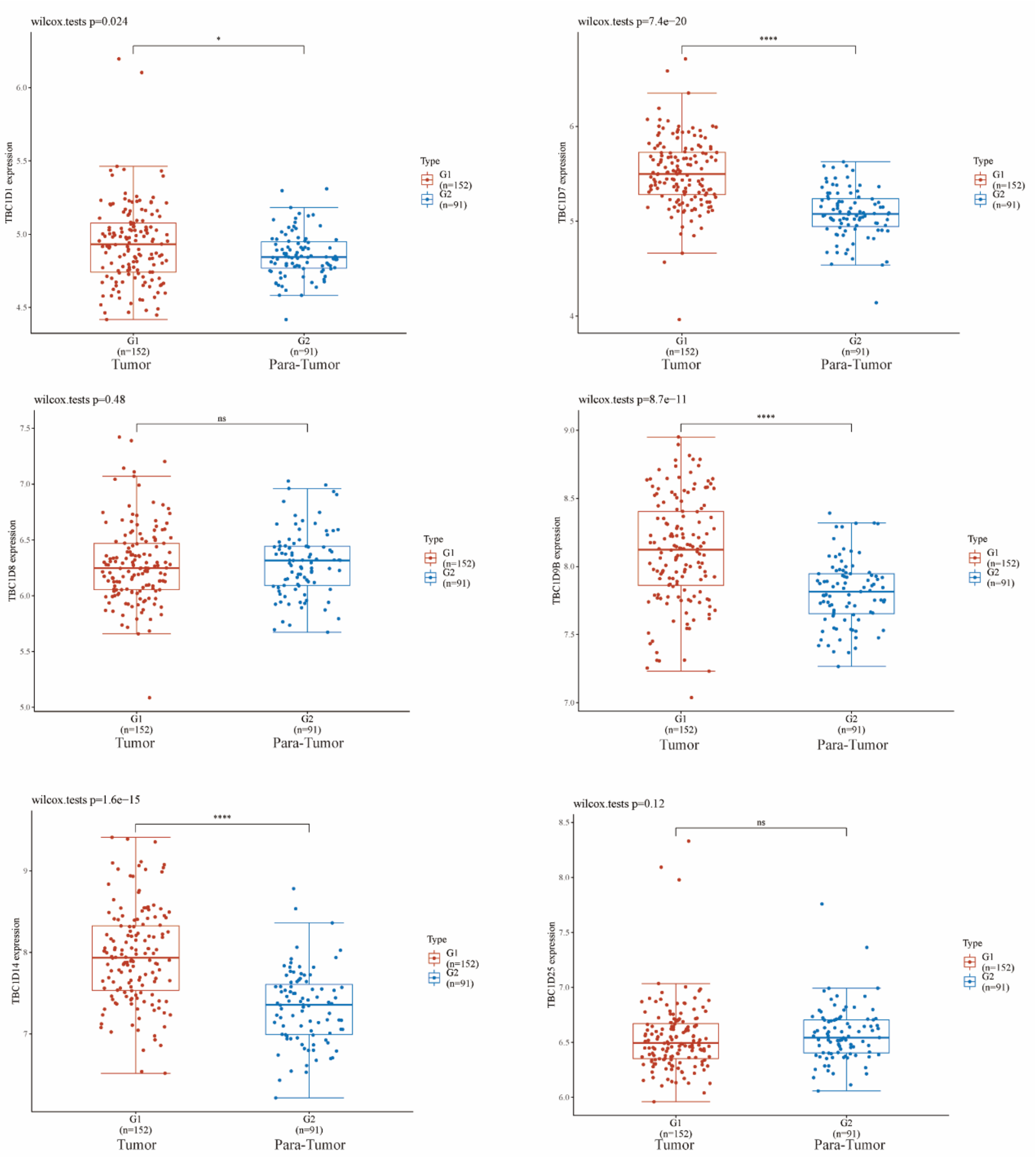

Supporting Figure 2. Transcript level of TBC1Ds in tumor and paratumor tissues from database of GSE102079.

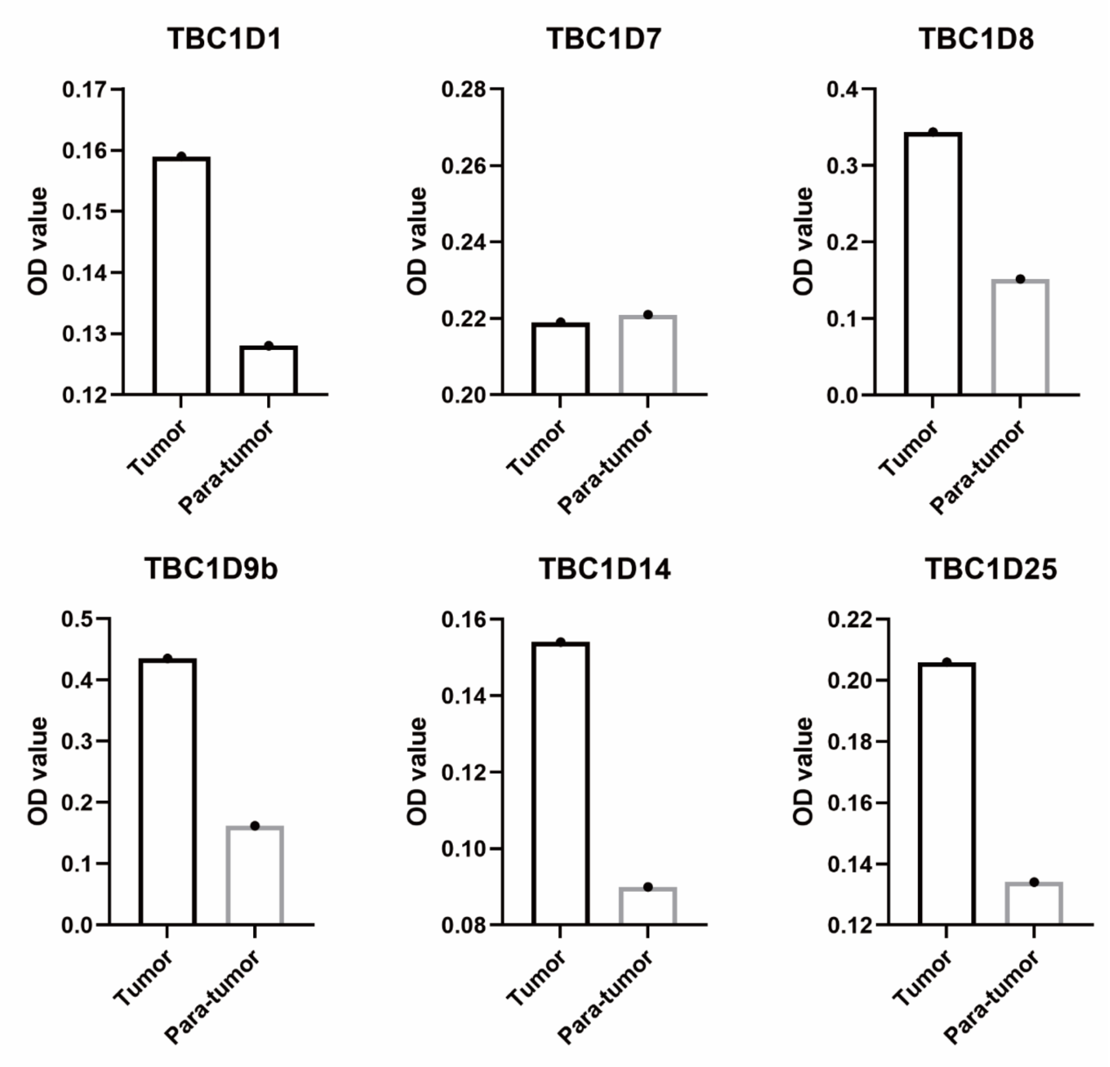

Supporting Figure 3. The IHC value of TBC1Ds in HCC and paratumor tissue.

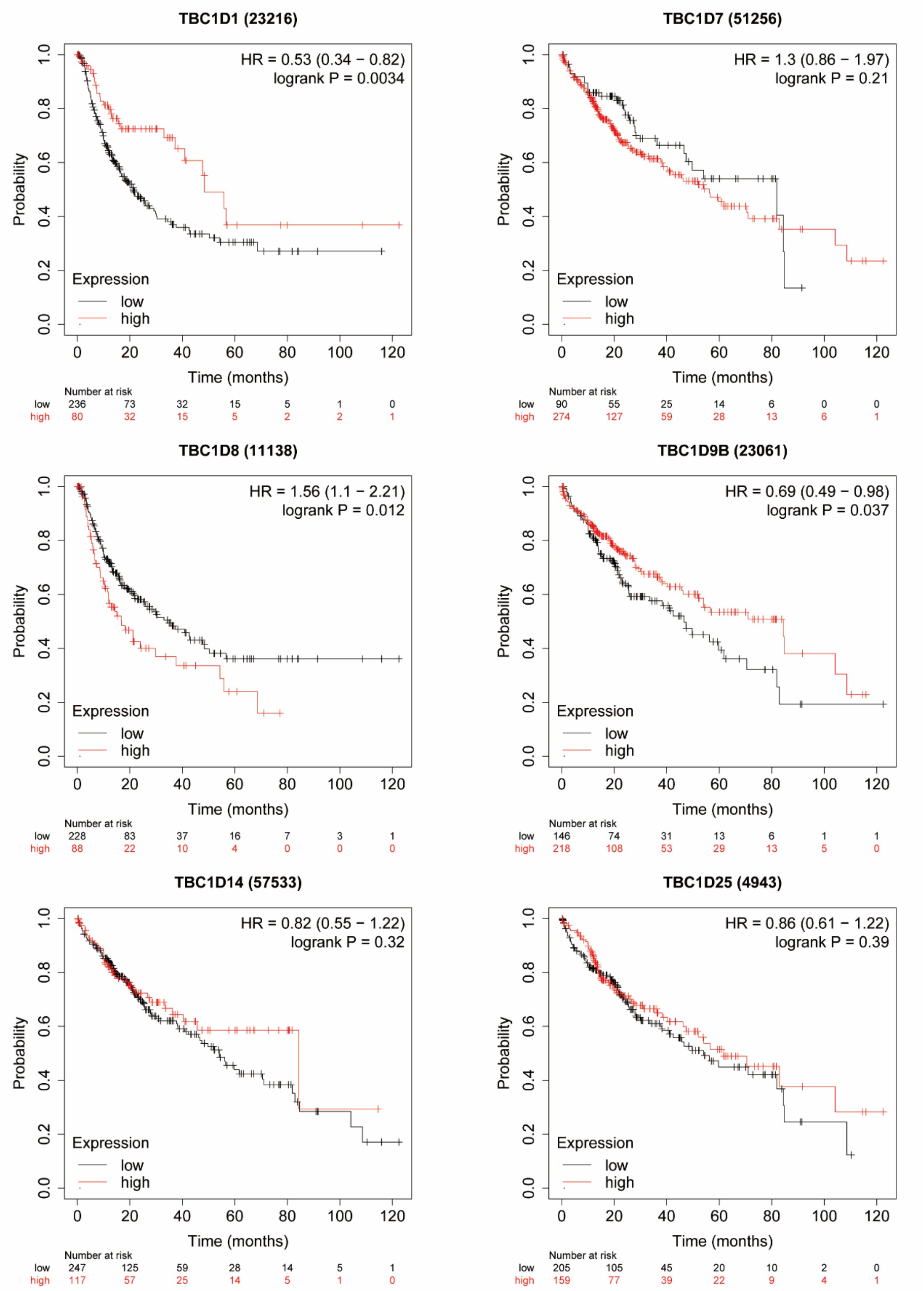

Supporting Figure 4. RFS analysis of the prognostic significance of TBC1Ds expressions based on the Kaplan-Meier Plotter database.

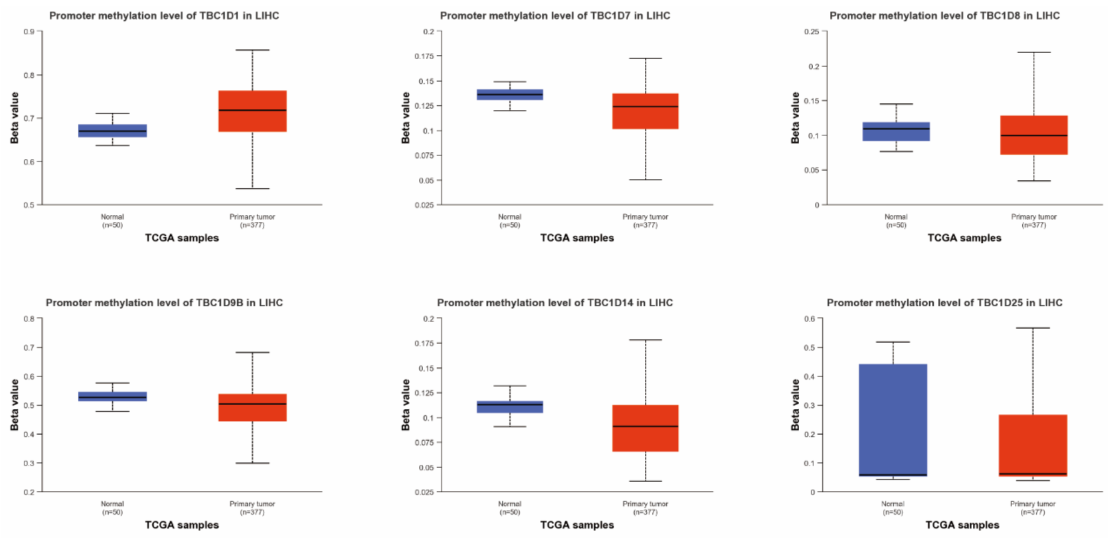

Supporting Figure 5. The promoter methylation levels of TBC1Ds in HCC according to UALCN database.

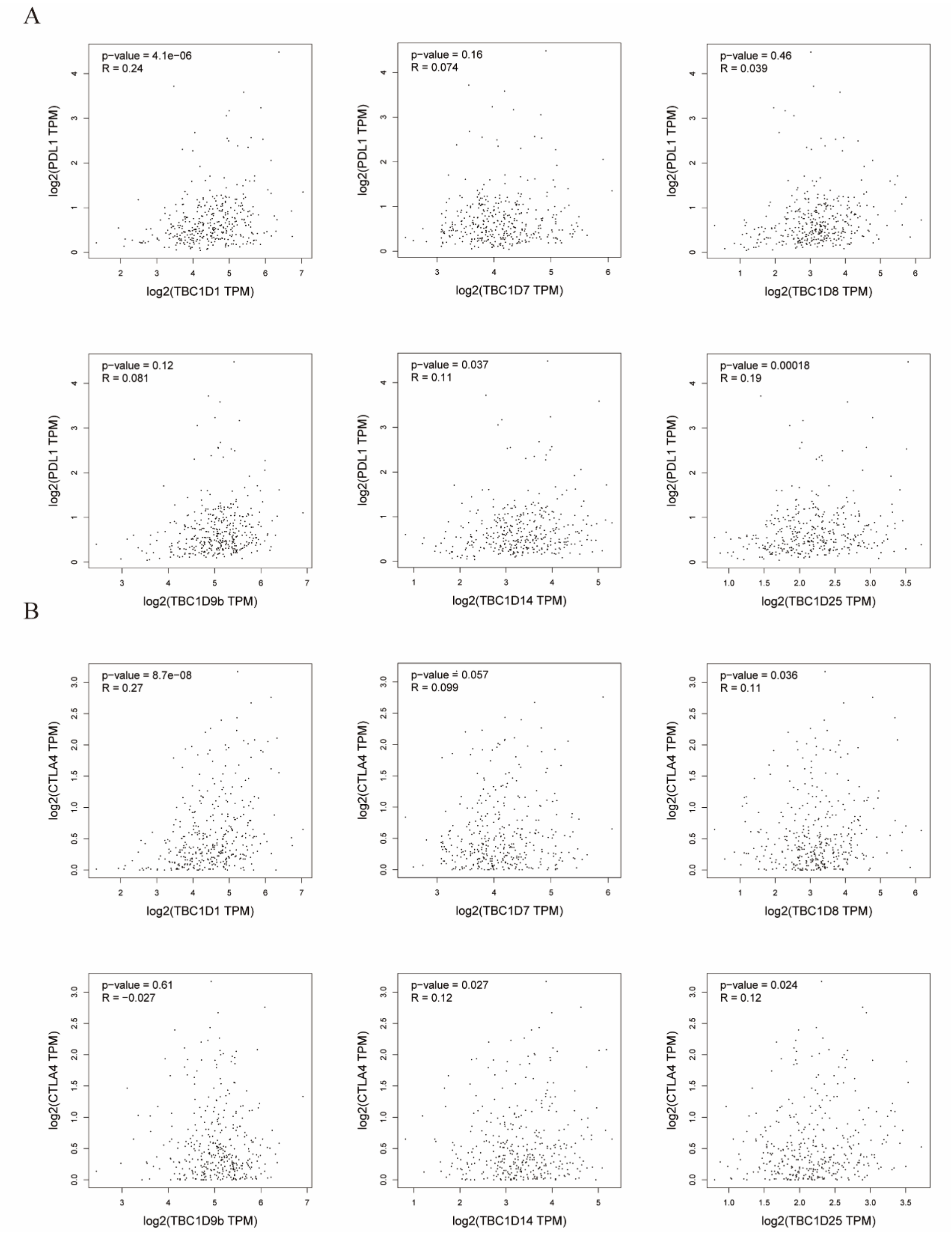

Supporting Figure 6. The correlation between TBC1Ds and the immune checkpoint receptors expression of PDL1 and CTLA4 in HCC.
